# Social media, education, and the rise of populist Euroscepticism

**DOI:** 10.1057/s41599-022-01317-y

**Published:** 2022-08-31

**Authors:** Piergiuseppe Fortunato, Marco Pecoraro

**Affiliations:** 1 United Nations Conference on Trade and Development (UNCTAD), Geneva, Switzerland; 2 Université de Neuchatel, Neuchâtel, Switzerland

**Keywords:** Politics and international relations, Economics, Cultural and media studies

## Abstract

This paper studies how the diffusion of skeptical or negative attitudes towards the European Union (EU) and the process of European integration relates to the new technologies of political communication, education, and their interaction. Using both European-wide and national surveys, we find a strong relationship between exposure to online political activity and Euroscepticism only among individuals with lower formal education. When distinguishing between different forms of online political activity it also finds that it is not the use of the internet per se that matters, but the specific use of social networks, like Twitter or Facebook, for obtaining information about politics. These results turn out to be robust to the use of instrumental variables intended to capture the speed of connection available and the relative easiness of using internet and social media.

## Introduction

**Fig. 1:**
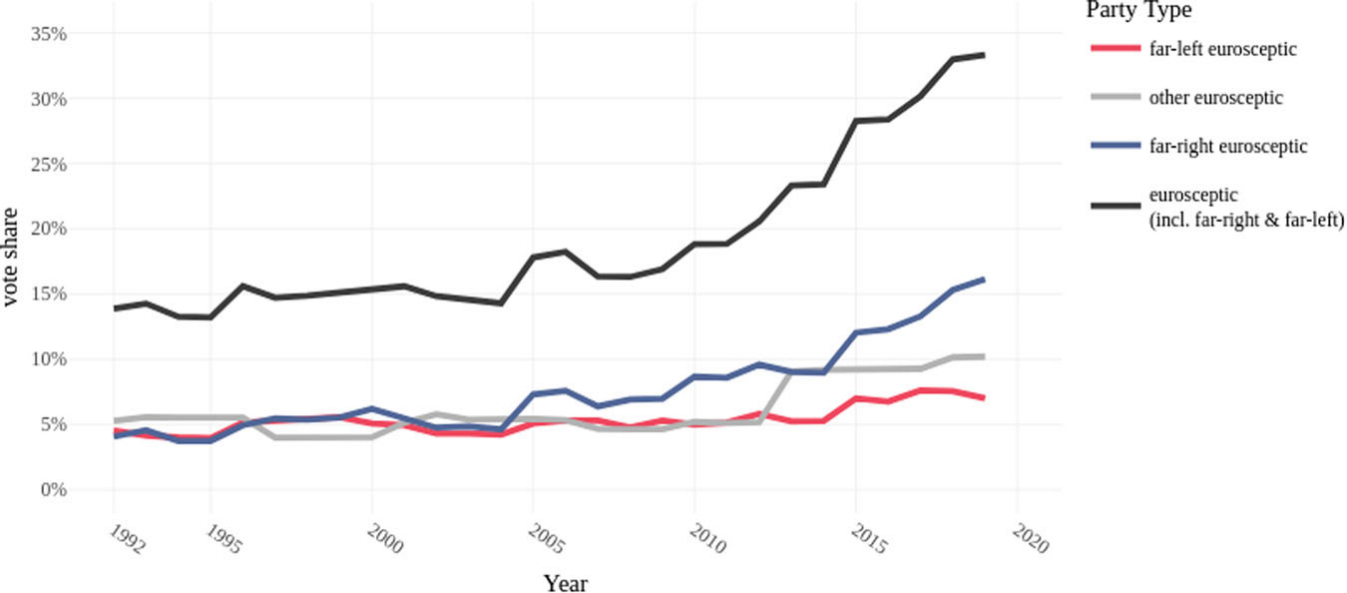
Share of Eurosceptic, far-right and far-left parties votes weighted by population size. Note: Data from ParlGov, European Countries; Classifications The PopuList, link: https://popu-list.org/.

The popular sentiment towards European institutions has changed enormously along the last three decades, especially after the turn of the millennium. The support for Eurosceptic parties has more than doubled since the early 1990s, with the combined vote share of parties proclaiming themselves Eurosceptic reaching almost the 34 percent in 2019. Figure 1 displays this explosive dynamic, driven mostly by anti-establishment parties on the far-right of the spectrum. The last time we saw a comparable diffusion of anti-establishment movements in the continent was in the mid-1930s, right before the collapse of the Weimer Republic (Dalio et al., 2017; Hopkin and Blyth, 2018).

The turn of the millennium also witnessed significant changes in the mass media space and in political communication technologies that affected the process of opinion formation. Citizens generally draw on information from the mass media to form political opinions. This is also the case for European integration. When the coverage is negative or framed in divisive terms, public support for European integration drops (Norris, 2000). The growing relevance of political information online and the emergence of “social” media have increased the exposition of voters to divisive messages (Maldonado, 2017). In some cases, as revealed recently by Frances Haugen, social media algorithms consciously privilege the most divisive content to amplify traffic on the networks. As a matter of fact, during the 2016 UK’s referendum campaign, the leave side dominated the day-to-day volume of tweets. Overall, along the last three weeks leading up to the vote, support for leaving on the platform outstripped support for remaining by a factor of four (Bauchowitz and Hänska, 2017). Similarly, from October 2018 to May 2019 before the EU parliamentary elections, eighty-five percent of all shared Facebook’s posts originated from all German political parties stem from AfD (Diehl et al., 2019).

Social media do not bear sole responsibility for the rapid spread of Euroscepticism. Animosity against European integration has been certainly aided by the worsening economic outlook and increasing inequality. The 2008–2009 global financial crisis and the following 2010–2011 European debt crisis resulted in job losses and significant drops in pensions, subsidies, and transfer payments, contributing to the progressive deterioration of the income distribution (European Parliament, 2015). This generated a diffuse sense of anxiety, especially among the most vulnerable sectors of the population, and increased the space for populist Eurosceptic political platforms designed to match the emerging demand of social justice (Algan et al., 2017).

Several studies have linked political support for Eurosceptic or populist movements to economic shocks and insecurity. Gozgor (2021) finds increases in total populism and right-wing populist voting behavior in Europe from 1980 to 2020 to be strictly related to increased global economic uncertainty. Higher penetration of Chinese imports has been found to be associated with support for Brexit in Britain and to the emergence of nationalist parties in continental Europe (Colantone and Stanig, 2016a, 2016b; Colantone and Stanig, 2018). Moreover in Sweden, increased labor-market insecurity has been linked empirically to the rise of the far-right Sweden Democrats (Dal et al., 2019).

Although online politics and social media are unlikely to be the sole, or even the main, driver of the diffusion of Eurosceptic sentiments, we posit that they can represent a key facilitator, especially among those less educated and less politically sophisticated individuals that might feel more exposed to the socioeconomic change brought about the European integration process (Betz, 2004) and that are on average more susceptible to divisive news (Schuck and de Vreese, 2006; and De Vreese et al., 2011).

Our hypothesis reflects in part the idea that large-scale socioeconomic change, induced by globalization and by integration processes, can produce winners and losers, and affect different groups differently, depending on the individual’s ability to adjust to (and take advantage of) the new situation. Individual ability to cope with large-scale change may depend critically on the amount of her or his *cultural capital*, which often refers to accumulated cultural knowledge, inherited values and beliefs individuals hold (Bourdieu, 1985; Throsby, 1999) and is likely to play a non-negligible role in shaping political opinions and behaviors. Since education tend to be a proxy for accumulated knowledge, cultural values and beliefs (see, e.g., Hainmueller and Hiscox, 2007), those individuals with low levels of education, and thus relatively low cultural capital, are more likely to be resistant to change and receptive to divisive ideas (Betz, 1994; Minkenberg, 2003; Rydgren, 2007).

Education is also essential for *social capital*; by promoting social interaction and reducing uncertainty about the behavior of others, it sustains civic norms and reinforces trust in institutions (Verba et al., 1995; Fortunato and Panizza, 2015). Low trust in institutions, in turn, has appeared to favor anti-establishment parties in Europe (Algan et al., 2017; Dustmann et al., 2017). Overall, therefore, education can play an important stabilizing role in modern democracies and, as such, smoothen divisive messages and clamp down on Euroscepticism.

Analyses of the 2016 referendum that paved the way to the exit of the United Kingdom from the EU, reveal a clear polarization of the vote along educational lines. A significant share of those with a lower level of education voted to leave, while citizens with the highest educational credentials voted to remain in vast majority (Hobolt, 2016). This educational divide is not a distinctive British feature. The data from the 8^th^ round of the European Social Survey (ESS), held in the same year of the Brexit referendum, reveal that the share of respondents that would vote for leaving the EU in case of a hypothetical referendum decreases consistently with the educational achievements. Almost half of the respondents having completed only the primary education cycle would be in favor of an exit from the EU while this figure drops down to around ten percent for respondents holding a master or an equivalent post-tertiary title.

We employ the 8th and 9th rounds of the ESS data to show that, in line with our hypothesis, exposure to political information online reinforces Eurosceptic preferences only among individuals with relatively low levels of education. For instance, we find that the correlation between exposure to political information online and being in favor for their own country leaving the EU is statistically significant only for individuals that underwent less that twelve years of education, roughly equivalent to the first two educational cycles in most European countries.

To study whether the specific use of social media matters, we further employ several rounds of the Italy’s Multipurpose Household Survey (MHS) that allows us to distinguish generic exposure to political information online and exposure mediated through social media. We find that it is not the use of internet per se that is associated with distrust in EU institutions but the specific use of social media by lower-educated individuals. Overall, our results confirm the existence of a strong association between sympathy for Eurosceptic ideas and exposure to online political activity documented by Galston (2018), Hendrickson and Galston (2017), and Alcott and Gentzkow (2017), among the others, but move one step forward characterizing the exact conditions under which exposure to online political activity matter.

In discussing our findings, we acknowledge that causality is hard to establish because our explanatory variables measuring the exposure to internet and social media are likely to be endogenous. Anti-EU activists and other politically motivated citizens might in fact be more prone to make use of internet (and social media) to get access to political (and politically biased) information and propaganda, and later share this material within their communities (Neumann and Gregorowictz, 2010). We deal with this issue following Campante et al. (2018) and instrumenting the exposure to online politics and social media using a series of variables intended to capture the speed of connection available to the respondent and therefore the relative easiness of using internet and social media to get access to political information. We show that our results are robust to the use of these instrumental variables.

The remaining of this paper is organized as follows. Section “Related literature and research question” defines Euroscepticism and presents some literature on its relations with populism in Europe. It also introduces our main research question on the non-trivial relations between online politics, education, and the diffusion of divisive messages as Euroscepticism. Section “Analysis” presents our analysis; it first introduces the datasets that we employ, then outlines our empirical strategy and reports the main findings. Section “Robustness analysis” presents the results of the robustness checks. Section “Discussion” discusses the relevance of our results while Section “Conclusions” offers some concluding remarks.

## Related literature and research question

While Euroscepticism can take different forms and shapes, it can be broadly defined as sceptical or negative attitudes towards the EU and the process of European integration. This definition dates to the seminal work of Paul Taggart, who suggests that Euroscepticism is best studied as an encompassing term that “expresses the idea of contingent, or qualified opposition, as well as incorporating outright and unqualified opposition to the process of European integration” (Taggart, 1998). It includes therefore both “soft” and “hard” forms of Euroscepticism, i.e., lack of trust in EU institutions and opposition to the process of European integration itself, respectively (Taggart and Szczerbiak, 2002). The former, accompanied by a strong criticism to the EUs’ current policies, seems nowadays a more common strategy among Eurosceptic parties (Dennison and Geddes, 2018).

This paper examines survey data on both trust in one of the chief EU institutions, the European parliament, and preferences for leaving the EU, i.e., outright opposition to the integration process. Euroscepticism is therefore the main object of our investigation while we do not directly examine populism. However, even if there is no necessary convergence between populism and Euroscepticism, the two phenomena have been strictly associated with recent times (Roodujin and van Kessel, 2019).

Although scholars may disagree on whether populism should be conceived as an ideology (Mudde, 2004), a discourse (Hawkins, 2010), or a style (Moffitt, 2016), many share the view that it revolves around the idea of an antagonistic relationship between the “good people” and the ‘evil elite’ articulated through emotionally charged communication strategies (de Vreese et al., 2018).

According to the influential definition of Cas Mudde, populism can be seen as “a thin-centred ideology that considers society to be ultimately separated into two homogenous and antagonistic groups, “the pure people” versus “the corrupt elite,” and which argues that politics should be an expression of the volonté générale (general will) of the people” (Mudde, 2004, p. 543). Part of the strength of such a political logic is that it involves an appeal to the entirety of the political community against a common enemy, and in particular against unresponsive political elites (Laclau, 2005; Gerbaudo, 2018).

Populists have therefore many reasons to oppose the EU, which can be pictured as a technocratic and elite-driven organization, far from the interests and the needs of ordinary citizens. Populists have also an easy time denouncing the complex and somehow opaque European decision-making processes, seen as a spanner preventing the implementation of the general will. Indeed, considering the populist hatred of backroom deals, shady compromises and complicated technicalities, Canovan (1999) has portraited EU politics as a “sitting duck” for populist attacks.

Criticism of European integration is not always framed in a populist manner, i.e., by referring to the antagonism between “people” and “elites”. Non-populist Eurosceptic discourses, for example, could be attributed to early Green parties’ criticism of the EU project (seen as potentially insufficiently global, too neoliberal, and non-environmental at that time), but Green parties have largely come to reconcile their politics with the important changes that took place in EU environmental regulation. At the same time, populists in Europe may not be particularly concerned with the issue of European integration (such as nationalist parties in Bulgaria or Slovakia). However, once again, as with non-populist Euroscepticism, the category is essentially a relatively small and diminishing number (Pirro and Taggart, 2018). In practice, therefore, populism and Euroscepticism have often been found in a close relationship, and the multiple shocks that hit the EU along the last decade have intensified the level of partisan competition and reinforced the Eurosceptic profile of populist movements (Alonso-Muñoz and Casero-Ripolles, 2020).

The strict association between populism and Euroscepticism is clearly reflected in the data collected by Rooduijn et al. (2019) that consider all the European parties that can be classified as populist, far right, far left, and Eurosceptic, and obtained at least 2 percent of the vote in at least one national parliamentary election since 1998. A clear overlap between Eurosceptic and populist movements emerges, with over three quarter of populist movements (both on the right and on the left of the political spectrum) also classified as Eurosceptic. In fact, while more attention has been devoted in the literature to how right-wing populists in Europe challenge the consensus on the benefits of European integration, left-wing critique to the EU based upon how communitarian institutions failed to protect Europe socially and put economic integration and prosperity ahead of people’s lives, cannot be dismissed (Eklundh, 2021).

Recent literature (see, e.g., Bergbauer et al., 2019), suggests the electoral successes collected by Eurosceptic and populist movements after the turn of the millennium can be partly explained by their ability to leverage salient issues, as immigration or unemployment, by offering radical and divisive alternatives that resonate with citizens’ fears and concerns. This feature can help us understanding the impact that the emergence of online political propaganda and social media might have add on political dynamics in Europe. There is in fact an emerging consensus in the cognitive and social sciences on the effects that the growing importance of political activities mediated through the internet, especially through social media, can have on partisan divisiveness. Several recent contributions find that exposure to political information online plays often an influential role in fostering the diffusion of divisive ideas, and aids polarization and political sectarism (Barret et al., 2021; Van Bavel et al., 2021; Finkel et al., 2020).

In a randomized experiment, Allcott et al. (2020) find that deactivating Facebook for the four weeks before the 2018 US midterm election and reducing online activity while increasing offline activities such as watching TV alone and socializing with family and friends, led to a significant reduction of both factual news knowledge and political polarization and to an increased subjective well-being. It did not reduce divisiveness based strictly on party identity, however. This is consistent with the view that people are seeing political content on social media that does tend to make them more upset, angrier at the other side and more likely to develop stronger and divisive views on specific issues.

It is the very design of the automated systems that run the platforms the main responsible for the amplification of divisive content. Social media technology employs popularity-based algorithms that tailor content to maximize user engagement thereby generating self-reinforcing feedback loops. Maximizing engagement in turn increases polarization, especially within homogeneous networks or groupings of like-minded users. Levy (2021) find that Facebook’s content-ranking algorithm may limit users’ exposure to news outlets offering viewpoints contrary to their own—and thereby increase polarization.

The consequences of this heightened partisan animosity include the unprecedented diffusion of conspiracy theories, an increase in political violence and the erosion of trust in elections and in traditional democratic institutions. In Europe, the consolidation of digital media aided a massive circulation of populist messages that question the political legitimacy of the European Union and diffuse mistrust in its chief institutions (Alonso-Muñoz and Casero-Ripolles, 2020).^1^ Several studies document the extensive use of social media propaganda by part of Eurosceptic movements before and after Brexit (see e.g., Galpin and Hans-Jörg, 2017; Zappettini and Maccaferri, 2021).

But are the effects of exposure to Eurosceptic political propaganda online homogeneous across different social groups? This paper investigates whether low formal education, an individual characteristic commonly found to predict Euroscepticism, become a more potent driver of Eurosceptic beliefs when it co-exists with a reliance on social media as a news source. Our hypothesis is that education reduces exposition to negative feedback loops on social media since higher educated individuals are on average less sceptic towards the European integration project. Furthermore, highly educated individuals are likely to discern more easily between mainstream and false news (Guess et al., 2020).^2^

Recent empirical literature shows the existence of a clear correlation between educational level and preferences towards European integration. Across Europe, those with less formal education are consistently found to be more Eurosceptic than those with higher education (Hooghe and Marks, 2005; Lubbers and Scheepers, 2010), and this gap has significantly widened over time (Lubbers and Jaspers, 2011).

Many reasons have been called upon to explain this evidence (for a detailed literature review, see Hakhverdian et al., 2013). From a purely economic perspective, one of the seminal explanations of the phenomenon asserts that higher educated individuals are likely to be more favorable to integrated labor markets, and therefore less Eurosceptic, because they face less competition and insecurity (Gabel and Palmer, 1995).^3^ Analogously, cognitive, creative, and functional skills predominantly transmitted in formal education might enable individuals to remain flexible and to successfully interact in an internationalized environment (Rosenau et al., 2004). From a more sociological perspective, in a wide variety of national contexts and time periods, low formal education has been repeatedly shown to be a powerful predictor of ethnic exclusionism and nationalism. Inglehart and Baker (2000), for example, argue that through their education individuals acquire the ability to cope with abstract and extensive political communities such as the EU.

## Analysis

### The data

We start presenting the datasets that we employ; the 8th and 9th rounds of the European Social Survey and several iterations of ISTAT Multipurpose Survey of Italian Families on “Aspects of Everyday Life” (Indagine Multiscopo sulle Famiglie). The discussion of our empirical strategy and of the main results is the object of the next sub-section.

#### European Social Survey

The European Social Survey (ESS) is a multi-country survey that monitors changing public attitudes and values within Europe and develops a series of European social indicators, including attitudinal indicators. The survey covers at least 23 countries and over 40,000 individuals per round (see www.europeansocialsurvey.org). The key topics covered by the ESS include social trust; political interest and participation; socio-political orientations; social exclusion; national, ethnic and religious allegiances; climate change, energy security and energy preferences; welfare; human values; demographics and socioeconomics. More importantly for our aims, the survey also investigates the attitude towards the EU and, only from the 8th round on, it includes a series of questions on online political activity, to assess whether the respondent posted or shared anything about politics online, for example on blogs, via email or on social media.

To measure Euroscepticism, we use two specific questions as recorded in the 8th and 9th rounds of the ESS, which were collected in 2016 and 2018. The first question allows us to measure the level of trust in European Parliament from 0 (no trust) to 10 (full trust); the second question is interested on whether respondents declare themselves in favor for their own country leaving the EU. These questions were asked in the 17 EU countries that participated to the 2016 and 2018 rounds of the survey.^4^ While we keep the ordinal values from 0 to 10 for trust in European parliament, we construct the dummy variable EU exit equal to 1 if respondents would vote for his country to leave and 0 in the case of voting to remain member of European Union. The average levels of trust in European parliament correspond to 4.26 among the full ESS sample in Panel I of Table A.1 in the Supplementary Appendix and to 4.33 among the sample of workers in Panel II of the same table, but these average values are not statistically different at the 95% confidence level. In addition, the average share of respondents in favor of leaving the EU is 19 percent either based on the full ESS sample (Panel I) or on the sample of individuals in paid work (Panel II). The variance across countries is considerable (results not shown); residents from Ireland emerge as the least Eurosceptic (with a trust level in European parliament of at least 5 and around 8 percent of the population in favor of leaving the EU, on average) while on the other side of the spectrum we find the UK (at least 3.7 and around 40 percent, respectively).

The key correlates of Euroscepticism considered in our analysis are the level of education and exposure to politics online. The ESS contains detailed information on the number of years of education of the respondents. While we measure the exposure to politics online (labeled online politics below) with a dummy variable that takes value equal to 1 if the respondent declares to have posted or shared something about politics online, for example on blogs, via email or on social media such as Facebook or Twitter, during the last 12 months, and 0 otherwise. As shown in Table A.1, between one fifth of the respondents from the full sample (Panel I) and one quarter from the sample of employed (Panel II) have posted or shared something about politics online. In addition, on average, employed respondents appear to be more educated than those from the full sample (mean years of education is at least 13 in Panel I and more than 14 in Panel II), this difference being statistically significant at the 95% confidence level.

#### ISTAT multipurpose household survey

The second dataset that we employ is the ISTAT Multipurpose Household Survey on “Aspects of Everyday Life”, which covers the Italian permanent resident population in private households by interviewing a sample of 20,000 households and 50,000 people. The survey provides information on the citizens’ habits in different thematic areas, including school, work, family and social life, spare time, political and social participation, health and lifestyle.

Interestingly, the ISTAT survey includes not only questions on trust in major EU institutions and on online participation in politics, but also distinguishes between the use of social networks to acquire information about politics and online political activities not mediated through these networks (e.g., consultation of websites linked to traditional media or blogs). It therefore allows us to refine the analysis based on ESS data and to assess also the impact on attitudes toward the EU of exposure to social media versus traditional media internet platforms (newspapers, televisions, etc.). We consider the years ranging from 2013 to 2016 (the latest available).

The key outcome variable here is represented by trust in European Parliament that ranges between 0 and 10, with higher values being associated with higher trust in the EU Parliament. The average level of trust in European parliament over the period 2013–2016 is approximatively 3.75 in the sample of employed individuals (Panel B of Table A.2 in the Supplementary Appendix) and is slightly higher considering the full sample (Panel A of Table A.2 in the Supplementary Appendix), indicating that the average Italian is rather Eurosceptic. Attitudes towards the European parliament have deteriorated over the period 2013–2015 and then stabilized around its lowest value. The average level of trust among employed individuals was 3.90 in 2013, 3.75 in 2014, and 3.68 in 2015 and in 2016.

The ISTAT survey contains detailed information on the level of education of the respondents (i.e., the highest diploma achieved). It also offers the possibility of controlling for sex, age group, civil status, household type, and the urban dimension of the city of residence.

As anticipated above, with regards to the exposure to politics online, the survey distinguishes between acquiring information about politics through social networks, like Facebook or Twitter, and acquiring information about politics on internet but in other ways (e.g., through websites related to traditional media or blogs). This distinction allows us to investigate whether different ways of using internet in the political realm are associated with different attitudes towards the EU.

As shown in Table A.2, 23 percent of the respondents from the full sample (Panel I) are exposed to politics online, and about 40 percent of them rely on social media to get political information on internet. In parallel, the share of respondents exposed to politics online is higher among the sample of employed (Panel II), corresponding to 13 percent and 22 percent when exposure operates through social media and traditional websites, respectively. Moreover, among the full sample, half of the respondents have a compulsory education only (Panel I). Among the sample of employed (Panel II), the share of low-educated is much lower (31 percent) and the majority holds a high-school diploma as highest degree (47 percent).

### Econometric specification and results

In this sub-section, we use econometric methods to check whether the partial correlations between our proxies for Euroscepticism (i.e., low trust in EU parliament and preference for exit from EU), formal education and exposure to political information online (and their interactions) are consistent with our hypothesis.

#### Education, online politics, and Euroscepticism

We first consider ESS data and start by studying the cross-sectional correlation between Euroscepticism, exposure to online politics and education. To account for the qualitative nature of the observed dependent variables, we use ordered and binary probit models in which cross-sectional individual weights are incorporated to produce representative estimates of the surveyed population.

In estimating the relationship between Euroscepticism and our key explanatory variables, we control for the age, age squared, sex, and foreign-born status of the respondent, and civil status. Wilkinson (2018) observes that rural areas and smaller urban centers are increasingly uniform in terms of social conservatism and constitute the basis of support for anti-establishment movements in many western economies. We therefore also include dummy variables aimed at controlling for this dimension: whether the respondent is living in suburbs of big city, in a small city, in the countryside or in a village. We also use as a control variable the level of household income declared by the respondents and classified in deciles. All specifications include the 2018-round fixed effect and country fixed effects.

Columns 1, 3, and 5 in Panel A of Table 1 show that exposure to online politics is not significantly correlated with trust in European parliament when not controlling for possible interaction effects. In Panel B, the corresponding coefficient estimates are positive (at a significance level of 0.1 or less), indicating that exposure to online politics is positively and significantly correlated with the propensity to be in favor of leaving the EU. Our results also show that the propensity to be Eurosceptic (i.e., low trust in EU parliament or being in favor of leaving the EU) is associated negatively with years of education, meaning that more educated individuals tend to display more trust in European parliament and to disfavor the idea of leaving the EU.

**Table 1:** Probit regressions of EU exit on online politics interacted with years of education.

	Full sample				In paid work	
	(1)	(2)	(3)	(4)	(5)	(6)
*Panel A: Trust in European parliament*						
Online politics	0.010 (0.017)	−0.105 (0.067)	−0.003 (0.018)	−0.139* (0.075)	0.028 (0.022)	−0.237** (0.099)
Years of education	0.028*** (0.002)	0.026*** (0.002)	0.023*** (0.002)	0.021*** (0.002)	0.032*** (0.003)	0.028*** (0.003)
Online politics*education		0.008* (0.004)		0.009** (0.005)		0.018*** (0.006)
Control variables	yes	yes	yes	yes	yes	yes
Country fixed effects	yes	yes	yes	yes	yes	yes
Round fixed effect	yes	yes	yes	yes	yes	yes
Household income (deciles)	no	no	yes	yes	yes	yes
Observations	60,719	60,719	49,399	49,399	27,270	27,270
*Panel B: EU exit Online politics*	0.063** (0.026)	0.432*** (0.109)	0.079*** (0.029)	0.502*** (0.117)	0.070* (0.036)	0.603*** (0.153)
Years of education	−0.052*** (0.003)	−0.047*** (0.003)	−0.043*** (0.004)	−0.037*** (0.004)	−0.058*** (0.005)	−0.049*** (0.006)
Online politics*education		−0.026*** (0.008)		−0.030*** (0.008)		−0.037*** (0.010)
Control variables	yes	yes	yes	yes	yes	yes
Country fixed effects	yes	yes	yes	yes	yes	yes
Round fixed effect	yes	yes	yes	yes	yes	yes
household income (deciles)	no	no	yes	yes	yes	yes
Observations	56,256	56,256	46,366	46,366	25,666	25,666

European Social Survey, rounds 8 (2016) & 9 (2018), www.europeansocialsurvey.org.

Ordered Probit (Panels A) and Probit coefficient estimates (Panel B); linearized standard errors in parentheses (data are weighted). Significance: ***p < 0.01, **p < 0.05, *p < 0.10. The analyses are based on a sample includes individuals from the following countries: Austria, Belgium, Czechia, Finland, France, Germany, Hungary, Ireland, Italy, Lithuania, Netherlands, Poland, Portugal, Slovenia, Spain, Sweden, and the United Kingdom. The first dependent variable *trust in European parliament* in Panel A is an ordinal variable, ranging from 0 (no trust at all) to 10 (complete trust). The second dependent variable *EU exit* in Panel B is coded as follows: 1, in favor of leaving the European Union; 0, in favor of remaining a member of the European Union. *Online politics* is coded as follows: 1, the respondent posted or shared anything about politics online during the last 12 months; 0, otherwise. Control variables: Sex, age, age squared, marital status, foreign born, and urban level.

These initial results assume that the coefficient estimates on education and exposure to online politics are independent of each other. Our working hypothesis, however, suggests the existence of an interaction between these variables. We expect the correlation between exposure to online politics and Euroscepticism to be strengthened when looking at individuals with low formal education.

We test formally for the presence of an interaction between exposure to online politics and education by estimating the following model:

$$y_i^ \ast = \alpha + \beta \,{online}\,{politics}_i + \gamma \,E_i + \delta \,{online}\,{politics}_i \times E_i + {{{\boldsymbol{C}}}}_i\rho + \varepsilon _i.$$

*y*_i_* is the unobserved latent variable for attitudes towards the EU (which is tied to one or the other observed outcome of interest available in the ESS), and *online politics*_*i*_ is the dummy variable on the exposure to politics on internet, while *E*_*i*_ represents the level of education (measured in years) and *ε*_*i*_ is the error term with a standard normal distribution, for individual *i*. *C*_*i*_ is a vector containing the different control variables along with income deciles, the 2018-round fixed effet and country fixed effects, as discussed above.

Columns 2, 4, and 6 in Table 1 overall show a positive and significant association between exposure to online politics and Euroscepticism (or, put differently, a negative association with favorable attitudes towards the EU) after the introduction of the interaction term. Also, the existence of a negative association between education and Euroscepticism is confirmed. More interestingly, we find clear evidence of a positive (resp. negative) and significant coefficient estimate of the interaction term between exposure to online politics and education in Panel A (resp. in Panel B), suggesting that the interplay between these two factors is an important driver in shaping the attitudes towards the EU.

**Fig. 2:**
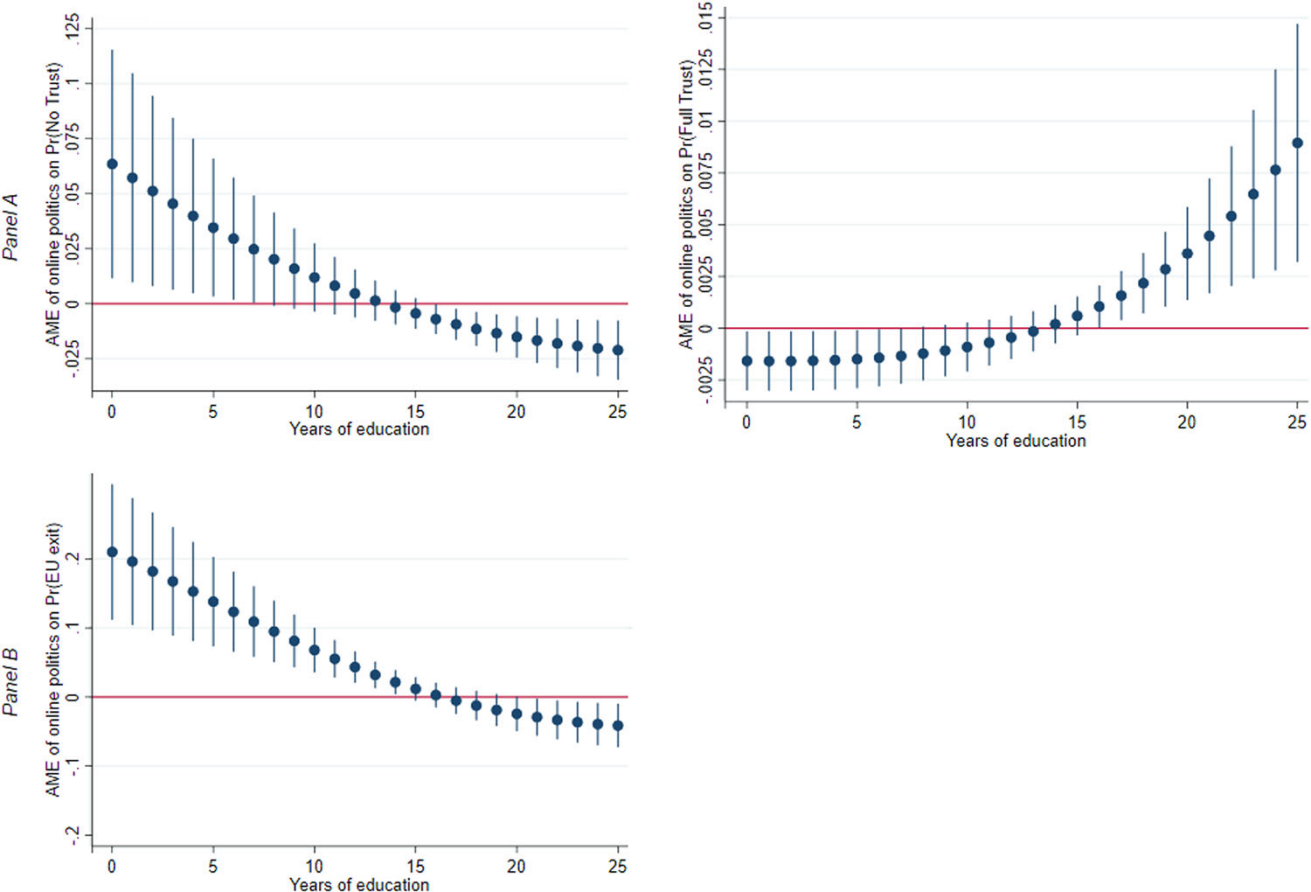
Average marginal effects of online politics by years of education. Calculation from the fourth column of Table 1. The average marginal effects are plotted with the 95 percent confidence intervals. The dependent and control variables are described in the note to Table 1.

Figure 2 uses the results of the fourth column in Table 1 to plot the average marginal effects of exposure to online politics on our two outcomes for attitudes towards the EU at different levels of education. The horizontal axis measures variations in the number of years of education attained. Given the ordinal nature of the outcome variable in Panel A, the average marginal effects are only computed for its lowest and highest level. Accordingly, column (1) in Fig. 2 presents the average marginal effects of exposure to online politics on Euroscepticism since the focus is on the lowest level of trust in European parliament (corresponding to the case of “no trust”), in addition to the likelihood of being in favor of an exit from the EU (Panel B). The reverse logic applies in column (2). Whatever the selected outcome, as shown in column (1), the average marginal effects are positive and statistically significant only for those individuals with relatively few years of education. Moreover, the average marginal effects of exposure to online politics on Euroscepticism are instead negative (and significant) for individuals with high educational attainment. The remaining figure in column (2) logically displays the opposite pattern.

#### The medium matters

Our main results are confirmed also when employing the ISTAT Multipurpose Household Survey. However, using this dataset allows us to move one step further and analyze the association between different forms of online activity and Euroscepticism.

We run ordered probit regressions with robust standard errors (clustered at the individual level) on the four separate rounds of the survey and on the complete dataset running from 2013 to 2016. The dependent variable in this set of regressions is represented by the level of trust in the EU parliament as divided in 11 ordered categories, ranging from no trust (0) to complete trust (10) and thus very similar in the spirit of the ESS question used in the previous sub-section. Put differently, the dependent variable can be thought of as a measure of propensity to exhibit positive attitudes toward the EU. The key independent variables are the level of education as divided in three categories (compulsory education, high-school diploma, and Bachelor and higher tertiary degrees) and the type of exposure to politics on internet. Since this latter variable takes three values, we construct two dummy variables (the reference category corresponding to the situation where the respondent does not use internet to get information about politics): the dummy online politics w/o social media equals 1 if the respondent makes use of internet to get information about politics but not through social media (0 otherwise) while the dummy online politics via social media equals 1 if the respondent makes use of internet to get information about politics through social media (0 otherwise). We control for all the individual characteristics mentioned above and always include region fixed effects and year fixed effects (when using the dataset pooled over all available years).

Columns 1 and 3 of Table 2 show that while the use of social media to get information on politics is always negatively and significantly correlated with trust in the EU parliament (especially when the sample is restricted to employed individuals), the simple use of internet to get access to information not mediated through social media is in general positively associated with trust in the parliament. This is particularly interesting since it highlights the specific role that social media play to diffuse anti-establishment and divisive ideas as opposed to the effect of the simple (increased) access to information enabled by the world wide web. These results also show that levels of education below tertiary degrees tend to be associated with lower trust in European institutions.

**Table 2:** Ordered probit regressions of trust in European parliament on online politics interacted with the level of education.

	Full sample		Sample of employed	
	(1)	(2)	(3)	(4)
Online politics w/o social media	0.048*** (0.008)	0.062*** (0.016)	0.045*** (0.011)	0.060*** (0.019)
Online politics via social media	−0.046*** (0.010)	−0.004 (0.020)	−0.083*** (0.013)	−0.012 (0.024)
High-school diploma	−0.183*** (0.009)	−0.169*** (0.012)	−0.211*** (0.011)	−0.192*** (0.016)
Compulsory school	−0.276*** (0.009)	−0.262*** (0.011)	−0.300*** (0.013)	−0.273*** (0.016)
Online politics w/o social media*h.s. diploma		−0.020 (0.019)		−0.009 (0.024)
Online politics w/o social media*compulsory school		−0.002 (0.025)		−0.026 (0.033)
Online politics via social media*h.s. diploma		−0.040* (0.024)		−0.076** (0.030)
Online politics via social media*compulsory school		−0.098*** (0.031)		−0.184*** (0.042)
Control variables	Yes	Yes	Yes	Yes
Italian region fixed effects	Yes	Yes	Yes	Yes
Year fixed effects	Yes	Yes	No	No
Observations	145,728	145,728	61,299	61,299

Multipurpose Survey on Households provided by https://www.istat.it.

Ordered Probit coefficient estimates; robust standard errors in parentheses (data are unweighted). Significance: ***p < 0.01, **p < 0.05, *p < 0.10. The analyses are based on the full sample (employed, unemployed, or out of the labor force) and on the sample of employed, pooled from 2013 to 2016, where individuals below 18 years old are excluded. The outcome variable *trust in European parliament* is an ordinal variable, ranging from 0 (no trust at all) to 10 (complete trust). The dummy variable *online politics via social media* measures whether an individual does inquire about politics online through social media, such as Facebook or Twitter. The dummy variable *online politics w/o social media* measures whether an individual does inquire about politics online without using social media. Control variables: Sex, age group, marital status, household type, and urban level.

Columns 2 and 4 of Table 2 confirm all the results even after the explicit introduction of interaction terms between different types of exposure to online politics and educational attainments. In line with our hypothesis, the coefficient estimated for the interaction term between the use of social media to get information about politics and the lowest educational attainment (completion of only compulsory schooling) is negative and in most cases strongly significant. Once again, this result suggests that exposure to social media among categories of lower-educated Italians is particularly effective in shaping attitudes towards Eurosceptic positions.

## Robustness analysis

Two main problems threaten the robustness of our results. The first is an omitted variable problem related to the potential endogeneity of education: if the level of education (or its interaction with exposure to online politics) is correlated with unobserved skills in computer and software use, related estimates from Table 2 would be plagued by an omitted variable bias. Indeed, low-educated individuals may exhibit poor knowledge about computer and internet, thus being more prone to misuse social networking sites to such an extent that they are unable to distinguish fake news from real news. Put differently, the possible correlation between low education and social media misuse may induce more exposure to Eurosceptic propaganda.

The first, second, fifth, and sixth columns of Table 3 show baseline estimates similar in the spirit of those displayed in the first four columns of Table 2: compulsory education and its interaction with online politics via social media are negatively associated with trust in the European parliament. To check the robustness of our results, we further control for indicators of self-assessed computer and software use that are available in this form only in 2015 and 2016.^5^*F* tests, reported in the third, fourth, seventh and eighth columns at the bottom of Table 3, indicate that their inclusion is jointly significant. At the same time, these additional regressions confirm our previous results. For instance, as presented in the fourth and eighth columns of the same table, the interactions between compulsory education and online politics via social media are still negative and significant when proxies for computer skills are taken into account.

**Table 3:** Ordered probit regressions of trust in European parliament on online politics interacted with the level of education, adding proxies for computer skills.

	Full sample				Sample of employed			
	w/o computer skills		With computer skills		w/o computer skills		With computer skills	
	(1)	(2)	(3)	(4)	(5)	(6)	(7)	(8)
Online politics w/o social media	0.024** (0.012)	0.027 (0.022)	0.010 (0.013)	0.019 (0.024)	0.024 (0.015)	0.026 (0.027)	0.014 (0.017)	0.023 (0.029)
Online politics via social media	−0.069*** (0.014)	−0.034 (0.027)	−0.089*** (0.015)	−0.054* (0.028)	−0.099*** (0.018)	−0.045 (0.032)	−0.106*** (0.020)	−0.055 (0.034)
High-school diploma	−0.190*** (0.012)	−0.182*** (0.017)	−0.173*** (0.013)	−0.163*** (0.018)	−0.218*** (0.015)	−0.211*** (0.022)	−0.211*** (0.016)	−0.202*** (0.023)
Compulsory school	−0.261*** (0.013)	−0.251*** (0.016)	−0.223*** (0.014)	−0.210*** (0.018)	−0.280*** (0.018)	−0.257*** (0.023)	−0.253*** (0.021)	−0.226*** (0.026)
Online politics w/o social media*h.s. diploma		−0.012 (0.027)		−0.019 (0.029)		0.015 (0.034)		0.005 (0.036)
Online politics w/o social media*compulsory school		0.032 (0.035)		0.024 (0.037)		−0.022 (0.047)		−0.037 (0.050)
Online politics via social media*h.s. diploma		−0.023 (0.032)		−0.019 (0.033)		−0.042 (0.041)		−0.037 (0.042)
Online politics via social media*compulsory school		−0.120*** (0.041)		−0.132*** (0.043)		−0.191*** (0.055)		−0.190*** (0.058)
Proxies for computer skills	No	No	Yes	Yes	No	No	Yes	Yes
Control variables	Yes	Yes	Yes	Yes	Yes	Yes	Yes	Yes
Italian region fixed effects	Yes	Yes	Yes	Yes	Yes	Yes	Yes	Yes
Year fixed effects	Yes	Yes	Yes	Yes	Yes	Yes	Yes	Yes
Test for joint significance of computer skills								
*F*-test			93.24***	93.58***			51.58***	52.17***
Observations	72,111	72,111	66,812	66,812	30,540	30,540	28,402	28,402

Multipurpose Survey on Households provided by https://www.istat.it.

Ordered Probit coefficient estimates; robust standard errors in parentheses (data are unweighted). Significance: ***p < 0.01, **p < 0.05, *p < 0.10. The analyses are based on the full sample (employed, unemployed, or out of the labor force) and the sample of employed workers, pooled over 2015 and 2016, where individuals below 18 years old are excluded. The outcome variable *trust in European parliament* is an ordinal variable, ranging from 0 (no trust at all) to 10 (complete trust). The dummy variable *online politics via social media* measures whether an individual does inquire about politics online through social media, such as Facebook or Twitter. The dummy variable *online politics w/o social media* measures whether an individual does inquire about politics online without using social media. The proxy variables for self-assessed computer skills are derived from various operations that were performed in the last 12 months. Control variables: Sex, age group, education level, marital status, household type, and urban level.

The main issue with the estimates presented in Table 2, however, relates to the potential endogeneity of our variables measuring the exposure to internet and social media. One can argue that anti-EU activists and other politically motivated citizens might be more prone to make use of internet (and social media) to get access to political (and politically biased) information and propaganda, and later share this material within their communities. Within the digitized public sphere, it is not only the leaders that communicate with followers, but also followers communicate among each other. Voters are therefore not simply pushed but they also pull by engaging actively in digital media or keeping up the digital bonding that expresses support for the populist movement (Neuman, 2016).

To address this issue, following Campante et al. (2018), we instrument the exposure to online politics and social media using a series of variables intended to capture the speed of connection available to the respondent and therefore the relative easiness of using internet and social media to get access to political information. We employ the following variables as instruments: availability of a DSL connection (yes/no), availability of a smartphone connection (yes/no), availability of a SIM/USB connection (yes/no) and availability of an ISDN connection (yes/no). These four binary variables are available only in 2014, 2015 and 2016, therefore for the IV analysis we are unable to use the information contained in the previous round of the MHS Survey.

Over the period 2014–2016, more than half of the respondents from the full sample use a DSL connection, about a quarter a smartphone connection, at least 13 percent a SIM/USB connection, and 2 percent an ISDN connection. These figures are more or less similar to those computed from the labor-force sample or the sample of employed respondents (results not shown).

We run an ordered probit IV with two first-stage probit regressions in which the dependent variables are exposure to politics online either through traditional websites (online politics w/o social media) or via social media (online politics via social media). As in the regressions of Table 3 we consider as a dependent variable the level of trust in the EU parliament. In order to investigate the differential impact of exposure to internet by education level, we split the sample between individuals having completed only compulsory education cycles (low educated) and those with high-school diploma, Bachelor or higher tertiary degrees (high educated). In our regressions we also introduce region fixed effects and control for sex, age cohort, civil status, household type and the urban dimension of the city of residence.

Table 4 displays the results obtained running our ordered probit IV specification on the full sample (employed, unemployed and out of the labor force) and compares the results of the instrumented analysis with the estimates of the standard model.^6^ We also tested specifications based on respondents in the labor-force sample or only employed respondents, leading to substantively equivalent results. On the whole, we find that the main results are robust to the use of instrumental variables. In particular, we find that the use of internet (without mediation via social media) does not play any role in influencing trust in the European parliament. On the contrary, getting information about politics on internet through social networks (like Facebook or Twitter) is negatively and significantly associated with trust in EU but only for low-educated individuals.

**Table 4:** Standard and IV Ordered Probit regressions of trust in European parliament on online politics by education.

	Compulsory education	Higher education	Compulsory education	Higher education
	Standard ordered probit		IV ordered probit	
	(1)	(2)	(3)	(4)
Online politics w/o social media	0.066*** (0.022)	0.064*** (0.011)	−0.048 (0.070)	0.009 (0.080)
Online politics via social media	−0.134*** (0.027)	−0.014 (0.013)	−0.216*** (0.065)	0.057 (0.049)
Control variables	Yes	Yes	Yes	Yes
Italian region fixed effects	Yes	Yes	Yes	Yes
Year fixed effects	Yes	Yes	Yes	Yes
*F*-test (dep. var.→ online politics w/o social media)			1284.04***	1253.75***
*F*-test (dep. var. → online politics via social media)			812.95***	1299.22***
Observations	53,192	55,004	52,917	54,910

Multipurpose Survey on Households provided by https://www.istat.it.

Standard and IV Ordered Probit coefficient estimates; robust standard errors in parentheses (data are unweighted). The IV ordered probit estimation involves two first-stage probit regressions. Significance: ***p < 0.01, **p < 0.05, *p < 0.10. The analyses are based on the full sample pooled over 2014, 2015 and 2016, where individuals below 18 years old are excluded. The outcome variable *trust in European parliament* is an ordinal variable, ranging from 0 (no trust at all) to 10 (complete trust). The dummy variable *online politics via social media* measures whether an individual does inquire about politics online through social media, such as Facebook or Twitter. The dummy variable *online politics w/o social media* measures whether an individual does inquire about politics online without using social media. The control variables are sex, age groups, married, household type, and urban level. The instrumental variables are DSL connexion (yes/no), smartphone connexion (yes/no), SIM/USB connexion (yes/no) and ISDN connexion (yes/no); these four variables are available in this form only in 2014, 2015, and 2016.

**Fig. 3:**
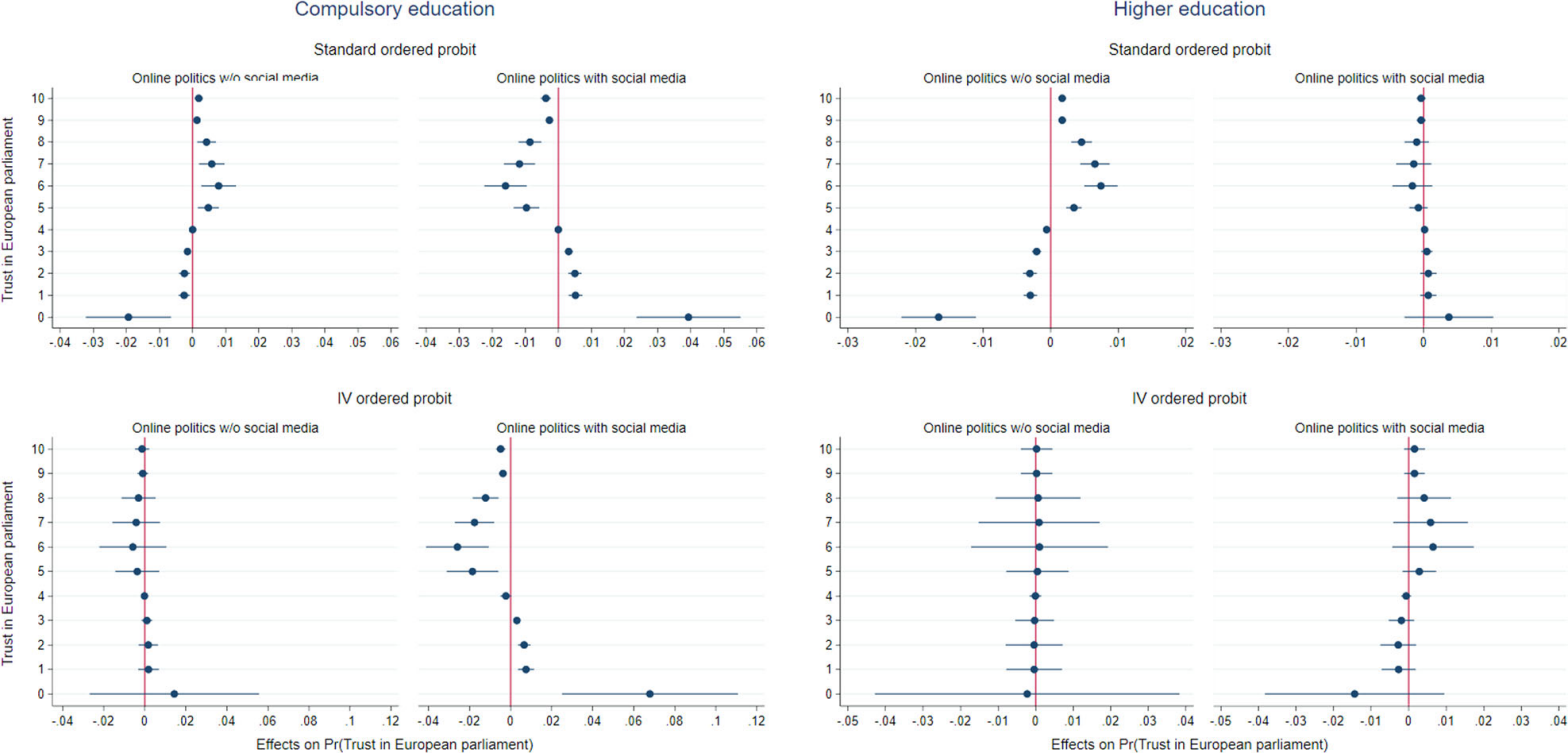
Average marginal effects of online politics by level of education. Note: Calculation from Table 4. The average marginal effects are plotted with the 95 percent confidence intervals.

Derived from Table 4, Fig. 3 allows us to visualize the average marginal effects of each type of internet use for political information on each category of trust in the European parliament. Among the low-educated, the average marginal effects of online political activity via social media are clearly negative for values of trust reaching at least the middle-scale position (these results hold for both the standard ordered probit and its IV version.) Interestingly, the average marginal effects estimated for the low-educated using the IV method tend to be higher than those estimated via the standard method. Put differently, standard estimates appear to underestimate the average marginal effects and could be considered as lower bounds of the true estimates.

## Discussion

Our results show how exposure to political information through “social media” has been closely associated with the diffusion of divisive ideas, like Euroscepticism, in Europe over the last decade. On the one hand this finding exposes the relevance of social media for the working of modern democracies and their political dynamics. On the other, it gives us important hints on the way in which certain political movements can make a strategic use of these media.

In line with our results, a recent literature review, while acknowledging the multi-faceted impact of social media and the complex nature of the phenomenon, concludes that, on balance, social media amplify political polarization, foment populism, and corrode trust in governments, news media, and institutions more in general (Lorenz-Spreen et al., 2022).

The current online ecosystem has been designed predominantly to capture user attention rather than to promote deliberate cognition and autonomous choice. As recently reported by former Facebook’s employee Frances Haugen, Facebook algorithms, as the ones of other common platforms, tend to privilege the most divisive content posted on the network. Such content is more frequently shared by users and foregrounding it maximizes traffic on the platform—and so turnover (Fortunato, 2021). This modus operandi is generating perverse incentives pushing even relatively moderate users to sharpen and polarize their content to obtain visibility.

Social media tend therefore to give more power and voice to the political extremes while reducing the power and voice of the moderate majority. The “Hidden Tribes” study, by the pro-democracy group More in Common, surveyed 8000 Americans in 2017 and 2018 and identified seven groups that shared beliefs and behaviors. It found that the two extreme groups, accounting for no more than the 14 percent of the sample were by far the most prolific group on social media (Hawkins et al., 2021).

The digitization of the public sphere has also changed how political actors and citizens relate to each other favoring a more direct and frequent communication between leaders and their base through different social networks. This social media-powered communication, in turn, by increasing the frequency of interactions and (allegedly) eliminating any filter may disproportionally favor populist movements that act as if a pure direct democracy were feasible by trying to create in voters the feeling of being directly addressed, and by offering emotionally charged political platforms to achieve this goal (Mudde, 2004; Maldonado, 2017). The Eurosceptic rhetoric, built around continuous and violent attacks to EU institutions, fits perfectly in such a scheme.

Recent research confirms that the disintermediation processes that characterize the social media system has fostered the spread of populist ideology in a fragmented form (Aalberg et al., 2016; Bracciale and Martella, 2017; Engesser et al., 2017; Wirth et al., 2016). A precise empirical assessment of the impact of social media diffusion on political outcomes is still missing, however. Our paper represents one of the first attempts in this direction. The use of survey data at the European and national level, in fact, allowed us to untangle the association between exposure to social media and certain types of political sentiments (i.e., attitude towards the EU), and to identify the categories more exposed to online propaganda.

In the future, it would be interesting to directly investigate the association between diffusion of social media use and voting patterns in both political elections and referenda. This would allow to assess how deep are the changes of opinion recorded in surveys (i.e., whether they are reflected in “hard” political preferences expressed through vote). It would also allow us to broaden the scope of the analysis and investigating more directly populism and the differences between far-left and far-right movements in their association with the internet sphere.

Another promising area for further investigation is the relationship between social media use and other socially relevant behaviors. One chief example in this area is attitude towards vaccination in the context of COVID-19. Recent literature suggests that critical factor in so many people choosing not to be vaccinated is their political views. Albrecht (2021), for example, explores the impacts of political views on vaccination rates in U.S. counties in 2021, and shows that in counties with a high percentage of Republican voters, vaccination rates were significantly lower. Much less explored, however, is the impact on vaccination rates of the interaction between political views and exposure to social media. Considering the results presented in our paper, as well as the related literature on the topic, this line of research seems particularly relevant for the future.

Finally, our research also highlights the potential instability of the existing institutional set-up in an era of social media diffusion and dominance. As recently put by Haidt (2022), “the norms, institutions, and forms of political participation that developed during the long era of mass communication are not going to work well now that technology has made everything so much faster and more multidirectional, and when bypassing professional gatekeepers is so easy […]. If we do not make major changes soon, then our institutions, our political system, and our society may collapse during the next major war, pandemic, financial meltdown, or constitutional crisis.” This represents indeed a call for policy oriented (normative) research aimed at reforming not only political institutions but also social media and web governance to smooth down polarization and sustain democratic coexistence (Lorenz-Spreen et al., 2020).

## Conclusions

A vast strand of literature has documented the association between low formal education and aversion towards the European integration project. More recent contributions from social and cognitive sciences suggest that recent changes in political communication strategies and the diffusion of political information online favored the diffusion of clear-cut and divisive messages, as abandoning the EU. This paper brings to the data the hypothesis that these two dimensions are not independent and tests whether the impact of exposure to political information online depends on the level of education of the citizens.

The paper examines how education, different uses of the internet to acquire information about politics, and their interactions, correlate with the diffusion Euroscepticism and distrust in EU institutions. Our results show that: (i) low education is associated with a higher propensity to be in favor of leaving the EU and to exhibit a lower trust in its institutions; and (ii) the interaction between education and exposure to online political activities is always negatively and significantly correlated with Euroscepticism. The latter finding indicates that the exposure to online politics is associated with Eurosceptic attitudes and distrust in the European institutions mainly for low-educated citizens. Furthermore, the different types of internet use, i.e., the acquisition of political information through social media or via more traditional sources of information on the web, play an important role. We find that (iii) it is not the use of internet per se that is associated with distrust in EU institutions but the specific use of social media for political activity by lower-educated Europeans.

## Supplementary information

Appendix

## Data Availability

The European Social Survey (ESS) data analyzed during the current study are available in the following public domain resources: https://www.europeansocialsurvey.org/data/conditions_of_use.html. We made use of: ESS Round 8: European Social Survey Round 8 Data (2016). Data file edition 2.2. Sikt—Norwegian Agency for Shared Services in Education and Research, Norway—Data Archive and distributor of ESS data for ESS ERIC. doi:10.21338/NSD-ESS8-2016. ESS Round 9: European Social Survey Round 9 Data (2018). Data file edition 3.1. Sikt—Norwegian Agency for Shared Services in Education and Research, Norway—Data Archive and distributor of ESS data for ESS ERIC. 10.21338/NSD-ESS9-2018. With respect to the second source of data analyzed in the study, we are bound by a confidentiality agreement with the Italian National Institute of Statistics (ISTAT) that does not allow us to preserve and share the Italian Multipurpose Household Survey data to the public. The only way to have access to these data is to request them from the ISTAT directly.

## Footnotes

1 In the case of Italy, Mazzoleni and Bracciale (2018) finds populism to be “endemic” in the online facebooksphere.

2 As discussed by Fortunato and Panizza (2015), education generally improve the functioning of democratic institutions by increasing informational flows and developing the cognitive skills that are necessary to effectively participate in a representative democracy.

3 As a matter of fact, increased exposure to immigrants appears to induce more negative attitudes towards immigration among low-educated workers or those working in economically declining sectors (e.g., Mayda, 2006; Pecoraro and Ruedin, 2016, 2020).

4 Not all EU countries are covered by the ESS. For more details and to see which countries took part in each ESS round, please consult https://www.europeansocialsurvey.org/about/participating_countries.html on the official ESS website.

5 The variables for self-assessed computer skills are derived from various operations that were performed in the last 12 months: transfer files between computers and/or other devices such as digital camera, cell phone, or MP3 player (yes/no), install software or applications (yes/no), change the settings of any software, including operating systems or security programs (yes/no), connect and install peripherals like printers or modems (yes/no), compress or zip files (yes/no), copy or move a file or folder (yes/no), use software for word processing like, e.g., Microsoft Word (yes/no), use “copy and paste” to copy or move information within a document (yes/no), create presentations or documents that include texts, images, graphics, tables (yes/no), use spreadsheets for calculation like, e.g., Microsoft Excel (yes/no), use the advanced functions of the spreadsheets for calculation to organize and analyze data like, e.g., sort, filter, use formulas, create graphics (yes/no), use software to edit photos, videos, audio files (yes/no) and write code in a programming language (yes/no).

6 The results presented in Table 4 also reports the values of the first-stage *F* statistics which are significant and relatively high, typically exceeding 10. Accordingly, the null hypothesis of weak instruments is always rejected using the *F* test on excluded instruments, casting out potential doubts on the validity of our instruments.
