# Supplementary material for: Social media, education, and the rise of populist Euroscepticism
Source: Humanit Soc Sci Commun. 2022 Aug 31;9(1):301. doi: 10.1057/s41599-022-01317-y (PMC9428866; doi:10.1057/s41599-022-01317-y)
Supplement: Supplementary file 1 — Appendix [file 41599_2022_1317_MOESM1_ESM.docx]

**APPENDIX**

Table A.1: Summary statistics for the key variables in the ESS

|  |  |  | Panel I: Full sample | | | Panel II: Sample of individuals in paid work | | |
| --- | --- | --- | --- | --- | --- | --- | --- | --- |
|  | Variable | | Mean | Linearized S.E. | Observations | Mean | Linearized S.E. | Observations |
|  | trust in European parliament | | 4.26 | 0.02 | 60,719 | 4.33 | 0.02 | 27,270 |
|  | online politics (yes/no) | | 0.20 | 0.00 | 60,719 | 0.26 | 0.00 | 27,270 |
|  | years of education | | 13.10 | 0.03 | 60,719 | 14.35 | 0.04 | 27,270 |
|  | EU exit (yes/no) | | 0.19 | 0.00 | 56,256 | 0.19 | 0.00 | 25,666 |
|  | online politics (yes/no) | | 0.20 | 0.00 | 56,256 | 0.25 | 0.00 | 25,666 |
|  | years of education | | 13.23 | 0.03 | 56,256 | 14.46 | 0.04 | 25,666 |

Source: European Social Survey, rounds 8 (2016) & 9 (2018), [www.europeansocialsurvey.org](http://www.europeansocialsurvey.org/).
Notes: Data are weighted. Summary statistics are based on samples pooled over 2016 and 2018. The full sample gathers respondents who are employed, unemployed or out of the labor force. *Trust in European parliament* is an ordinal variable, ranging from 0 (no trust at all) to 10 (complete trust). *EU exit* is coded as follows: 1, in favor of leaving the European Union; 0, in favor of remaining a member of the European Union. Included countries are: Austria, Belgium, Czechia, Finland, France, Germany, Hungary, Ireland, Italy, Lithuania, Netherlands, Poland, Portugal, Slovenia, Spain, Sweden, and the United Kingdom.

Table A.2: Summary statistics for the key variables in the MHS

|  |  |  | Panel I: Full sample | | | Panel II: Sample of employed individuals | | |
| --- | --- | --- | --- | --- | --- | --- | --- | --- |
|  | Variable | | Mean | Standard S.E. | Observations | Mean | Standard S.E. | Observations |
|  | | |  |  |  |  |  |  |
|  | trust in European parliament | | 3.78 | 0.01 | 145,728 | 3.75 | 0.01 | 61,299 |
|  | online politics w/o social media (yes/no) | | 0.14 | 0.00 | 145,728 | 0.22 | 0.00 | 61,299 |
|  | online politics via social media (yes/no) | | 0.09 | 0.00 | 145,728 | 0.13 | 0.00 | 61,299 |
|  | level of education | |  |  |  |  |  |  |
|  |  | bachelor and higher tertiary degrees | 0.13 | 0.00 | 145,728 | 0.21 | 0.00 | 61,299 |
|  |  | high school diploma | 0.37 | 0.00 | 145,728 | 0.47 | 0.00 | 61,299 |
|  |  | compulsory school | 0.50 | 0.00 | 145,728 | 0.31 | 0.00 | 61,299 |

Source: Multipurpose Survey on Households provided by [https://www.istat.it](https://www.istat.it/).
Notes: Data are unweighted. Summary statistics are based on samples pooled over 2013, 2014, 2015 and 2016, where individuals below 18 years old are excluded. The full sample gathers respondents who are employed, unemployed or out of the labor force. *Trust in European parliament* is an ordinal variable, ranging from 0 (no trust at all) to 10 (complete trust).

Table A.3.1: Trust in European parliament (Panel A)

|  | (1) | (2) | (3) | (4) | (5) | (6) |
| --- | --- | --- | --- | --- | --- | --- |
|  | Full sample | | | | In paid work | |
|  |  |  |  |  |  |  |
| online politics | 0.010 | -0.105 | -0.003 | -0.139* | 0.028 | -0.237** |
|  | (0.017) | (0.067) | (0.018) | (0.075) | (0.022) | (0.099) |
| years of education | 0.028*** | 0.026*** | 0.023*** | 0.021*** | 0.032*** | 0.028*** |
|  | (0.002) | (0.002) | (0.002) | (0.002) | (0.003) | (0.003) |
| online politics*education |  | 0.008* |  | 0.009** |  | 0.018*** |
|  |  | (0.004) |  | (0.005) |  | (0.006) |
| women | 0.074*** | 0.074*** | 0.092*** | 0.092*** | 0.101*** | 0.102*** |
|  | (0.012) | (0.012) | (0.014) | (0.014) | (0.018) | (0.018) |
| age | -0.048*** | -0.048*** | -0.049*** | -0.049*** | -0.042*** | -0.042*** |
|  | (0.002) | (0.002) | (0.002) | (0.002) | (0.005) | (0.005) |
| age squared | 0.000*** | 0.000*** | 0.000*** | 0.000*** | 0.000*** | 0.000*** |
|  | (0.000) | (0.000) | (0.000) | (0.000) | (0.000) | (0.000) |
| married | 0.339*** | 0.338*** | 0.362*** | 0.360*** | 0.367*** | 0.364*** |
|  | (0.026) | (0.026) | (0.029) | (0.029) | (0.036) | (0.036) |
| foreign born | 0.083*** | 0.084*** | 0.037** | 0.038** | 0.075*** | 0.076*** |
|  | (0.014) | (0.014) | (0.016) | (0.016) | (0.020) | (0.020) |
| suburbs of big city | -0.042* | -0.042* | -0.062** | -0.061** | -0.097*** | -0.096*** |
|  | (0.025) | (0.025) | (0.028) | (0.028) | (0.036) | (0.036) |
| small city | -0.068*** | -0.068*** | -0.088*** | -0.087*** | -0.107*** | -0.106*** |
|  | (0.021) | (0.021) | (0.023) | (0.023) | (0.030) | (0.030) |
| village | -0.120*** | -0.120*** | -0.150*** | -0.150*** | -0.199*** | -0.198*** |
|  | (0.021) | (0.021) | (0.023) | (0.023) | (0.029) | (0.029) |
| home in countryside | -0.135*** | -0.136*** | -0.167*** | -0.167*** | -0.173*** | -0.173*** |
|  | (0.035) | (0.035) | (0.039) | (0.039) | (0.053) | (0.053) |
| country fixed effects | yes | yes | yes | yes | yes | yes |
| round fixed effect | yes | yes | yes | yes | yes | yes |
| household income (deciles) | no | no | yes | yes | yes | yes |
|  |  |  |  |  |  |  |
| Observations | 60,719 | 60,719 | 49,399 | 49,399 | 27,270 | 27,270 |
|  |  |  |  |  |  |  |

Source: European Social Survey, rounds 8 (2016) & 9 (2018), [www.europeansocialsurvey.org](http://www.europeansocialsurvey.org/)
Notes: Ordered Probit coefficient estimates; linearized standard errors in parentheses (data are weighted). Significance: *** p<0.01, ** p<0.05, * p<0.10. Included countries are Austria, Belgium, Czechia, Finland, France, Germany, Hungary, Ireland, Italy, Lithuania, Netherlands, Poland, Portugal, Slovenia, Spain, Sweden, and the United Kingdom. The dependent variable *trust in European parliament* is an ordinal variable, ranging from 0 (no trust at all) to 10 (complete trust). *Online politics* is coded as follows: 1, the respondent posted or shared anything about politics online during the last 12 months; 0, otherwise.

Table A.3.2: EU exit (Panel B)

|  | (1) | (2) | (3) | (4) | (5) | (6) |
| --- | --- | --- | --- | --- | --- | --- |
|  | Full sample | | | | In paid work | |
|  |  |  |  |  |  |  |
| online politics | 0.063** | 0.432*** | 0.079*** | 0.502*** | 0.070* | 0.603*** |
|  | (0.026) | (0.109) | (0.029) | (0.117) | (0.036) | (0.153) |
| years of education | -0.052*** | -0.047*** | -0.043*** | -0.037*** | -0.058*** | -0.049*** |
|  | (0.003) | (0.003) | (0.004) | (0.004) | (0.005) | (0.006) |
| online politics*education |  | -0.026*** |  | -0.030*** |  | -0.037*** |
|  |  | (0.008) |  | (0.008) |  | (0.010) |
| women | -0.118*** | -0.118*** | -0.126*** | -0.126*** | -0.158*** | -0.160*** |
|  | (0.020) | (0.020) | (0.022) | (0.022) | (0.030) | (0.030) |
| age | 0.050*** | 0.050*** | 0.051*** | 0.051*** | 0.054*** | 0.054*** |
|  | (0.003) | (0.003) | (0.004) | (0.004) | (0.008) | (0.008) |
| age squared | -0.000*** | -0.000*** | -0.000*** | -0.000*** | -0.001*** | -0.001*** |
|  | (0.000) | (0.000) | (0.000) | (0.000) | (0.000) | (0.000) |
| married | -0.218*** | -0.214*** | -0.203*** | -0.199*** | -0.197*** | -0.192*** |
|  | (0.039) | (0.039) | (0.042) | (0.042) | (0.055) | (0.055) |
| foreign born | -0.133*** | -0.135*** | -0.066*** | -0.068*** | -0.082*** | -0.084*** |
|  | (0.021) | (0.021) | (0.025) | (0.025) | (0.031) | (0.031) |
| suburbs of big city | 0.063 | 0.063 | 0.068 | 0.067 | 0.116* | 0.112* |
|  | (0.043) | (0.043) | (0.047) | (0.047) | (0.060) | (0.060) |
| small city | 0.099*** | 0.098*** | 0.114*** | 0.112*** | 0.159*** | 0.156*** |
|  | (0.033) | (0.033) | (0.036) | (0.036) | (0.047) | (0.047) |
| village | 0.123*** | 0.122*** | 0.153*** | 0.151*** | 0.191*** | 0.187*** |
|  | (0.033) | (0.033) | (0.036) | (0.036) | (0.047) | (0.047) |
| home in countryside | 0.183*** | 0.185*** | 0.232*** | 0.234*** | 0.240*** | 0.239*** |
|  | (0.053) | (0.053) | (0.057) | (0.057) | (0.077) | (0.077) |
| country fixed effects | yes | yes | yes | yes | yes | yes |
| round fixed effect | yes | yes | yes | yes | yes | yes |
| household income (deciles) | no | no | yes | yes | yes | yes |
|  |  |  |  |  |  |  |
| Observations | 56,256 | 56,256 | 46,366 | 46,366 | 25,666 | 25,666 |
|  |  |  |  |  |  |  |

Source: European Social Survey, rounds 8 (2016) & 9 (2018), [www.europeansocialsurvey.org](http://www.europeansocialsurvey.org/)
Notes: Probit coefficient estimates; linearized standard errors in parentheses (data are weighted). Significance: *** p<0.01, ** p<0.05, * p<0.10. Included countries are Austria, Belgium, Czechia, Finland, France, Germany, Hungary, Ireland, Italy, Lithuania, Netherlands, Poland, Portugal, Slovenia, Spain, Sweden, and the United Kingdom. The dependent variable *EU exit* is coded as follows: 1, in favor of leaving the European Union; 0, in favor of remaining a member of the European Union. *Online politics* is coded as follows: 1, the respondent posted or shared anything about politics online during the last 12 months; 0, otherwise.

Table A.4.1

|  | (1) | (2) | (3) | (4) | (5) | (6) | (7) | (8) | (9) | (10) |
| --- | --- | --- | --- | --- | --- | --- | --- | --- | --- | --- |
|  | 2013 to 2016 | | 2013 | | 2014 | | 2015 | | 2016 | |
|  |  |  |  |  |  |  |  |  |  |  |
| online politics w/o social media | 0.048*** | 0.062*** | 0.082*** | 0.121*** | 0.058*** | 0.073** | 0.041** | 0.040 | 0.006 | 0.018 |
|  | (0.008) | (0.016) | (0.016) | (0.032) | (0.017) | (0.032) | (0.017) | (0.031) | (0.017) | (0.032) |
| online politics via social media | -0.046*** | -0.004 | -0.005 | 0.056 | -0.033 | 0.006 | -0.026 | 0.033 | -0.110*** | -0.097** |
|  | (0.010) | (0.020) | (0.021) | (0.041) | (0.022) | (0.042) | (0.020) | (0.038) | (0.019) | (0.038) |
| high school diploma | -0.183*** | -0.169*** | -0.188*** | -0.164*** | -0.163*** | -0.145*** | -0.211*** | -0.199*** | -0.168*** | -0.163*** |
|  | (0.009) | (0.012) | (0.017) | (0.024) | (0.018) | (0.024) | (0.017) | (0.023) | (0.017) | (0.024) |
| compulsory school | -0.276*** | -0.262*** | -0.305*** | -0.275*** | -0.276*** | -0.266*** | -0.275*** | -0.263*** | -0.247*** | -0.238*** |
|  | (0.009) | (0.011) | (0.018) | (0.023) | (0.018) | (0.023) | (0.018) | (0.023) | (0.018) | (0.023) |
| online politics w/o social media*h.s. diploma |  | -0.020 |  | -0.029 |  | -0.037 |  | -0.001 |  | -0.030 |
|  |  | (0.019) |  | (0.038) |  | (0.040) |  | (0.038) |  | (0.040) |
| online politics w/o social media*compulsory school |  | -0.002 |  | -0.105** |  | 0.033 |  | 0.027 |  | 0.030 |
|  |  | (0.025) |  | (0.050) |  | (0.051) |  | (0.048) |  | (0.051) |
| online politics via social media*h.s. diploma |  | -0.040* |  | -0.073 |  | -0.052 |  | -0.067 |  | 0.019 |
|  |  | (0.024) |  | (0.049) |  | (0.050) |  | (0.046) |  | (0.045) |
| online politics via social media*compulsory school |  | -0.098*** |  | -0.089 |  | -0.057 |  | -0.125** |  | -0.122** |
|  |  | (0.031) |  | (0.063) |  | (0.066) |  | (0.060) |  | (0.057) |
| women | 0.046*** | 0.046*** | 0.030*** | 0.029*** | 0.057*** | 0.058*** | 0.048*** | 0.047*** | 0.052*** | 0.052*** |
|  | (0.006) | (0.006) | (0.011) | (0.011) | (0.011) | (0.011) | (0.011) | (0.011) | (0.011) | (0.011) |
| 25-39 | -0.189*** | -0.191*** | -0.172*** | -0.175*** | -0.136*** | -0.137*** | -0.244*** | -0.247*** | -0.207*** | -0.209*** |
|  | (0.012) | (0.012) | (0.024) | (0.024) | (0.024) | (0.024) | (0.025) | (0.025) | (0.025) | (0.025) |
| 40-54 | -0.152*** | -0.153*** | -0.089*** | -0.091*** | -0.127*** | -0.127*** | -0.222*** | -0.224*** | -0.175*** | -0.174*** |
|  | (0.013) | (0.013) | (0.025) | (0.025) | (0.025) | (0.025) | (0.026) | (0.026) | (0.026) | (0.026) |
| 55-64 | -0.147*** | -0.149*** | -0.069** | -0.072** | -0.109*** | -0.109*** | -0.208*** | -0.210*** | -0.207*** | -0.208*** |
|  | (0.014) | (0.014) | (0.028) | (0.028) | (0.028) | (0.028) | (0.028) | (0.029) | (0.028) | (0.028) |
| 65+ | -0.051*** | -0.053*** | -0.019 | -0.024 | -0.006 | -0.005 | -0.092*** | -0.094*** | -0.087*** | -0.089*** |
|  | (0.014) | (0.014) | (0.028) | (0.028) | (0.028) | (0.028) | (0.029) | (0.029) | (0.028) | (0.028) |
|  |  |  |  |  |  |  |  |  |  |  |
| (continued on next page) | | | | | | | | | | |
|  |  |  |  |  |  |  |  |  |  |  |
|  |  |  |  |  |  |  |  |  |  |  |
|  |  |  |  |  |  |  |  |  |  |  |
|  |  |  |  |  |  |  |  |  |  |  |
|  |  |  |  |  |  |  |  |  |  |  |
|  |  |  |  |  |  |  |  |  |  |  |
|  |  |  |  |  |  |  |  |  |  |  |
|  |  |  |  |  |  |  |  |  |  |  |
|  |  |  |  |  |  |  |  |  |  |  |
| Table A.4.1 (continued) | | | | | | | | | | |
|  |  |  |  |  |  |  |  |  |  |  |
| married | 0.042*** | 0.042*** | 0.026 | 0.027 | 0.033* | 0.033* | 0.085*** | 0.086*** | 0.025 | 0.024 |
|  | (0.010) | (0.010) | (0.019) | (0.019) | (0.019) | (0.019) | (0.019) | (0.019) | (0.019) | (0.019) |
| couple with children | -0.067*** | -0.067*** | -0.053** | -0.053** | -0.080*** | -0.080*** | -0.089*** | -0.089*** | -0.046** | -0.045** |
|  | (0.011) | (0.011) | (0.022) | (0.022) | (0.023) | (0.023) | (0.022) | (0.022) | (0.022) | (0.022) |
| couple without children | -0.063*** | -0.063*** | -0.011 | -0.011 | -0.087*** | -0.088*** | -0.118*** | -0.118*** | -0.039* | -0.038 |
|  | (0.012) | (0.012) | (0.024) | (0.024) | (0.024) | (0.024) | (0.024) | (0.024) | (0.024) | (0.024) |
| single-parent father | -0.049** | -0.049** | -0.065 | -0.064 | -0.131*** | -0.130*** | -0.014 | -0.015 | 0.008 | 0.008 |
|  | (0.021) | (0.021) | (0.044) | (0.044) | (0.040) | (0.040) | (0.044) | (0.044) | (0.042) | (0.042) |
| single-parent mother | -0.064*** | -0.064*** | -0.045* | -0.045* | -0.105*** | -0.105*** | -0.063*** | -0.063*** | -0.043* | -0.042* |
|  | (0.012) | (0.012) | (0.024) | (0.024) | (0.024) | (0.024) | (0.023) | (0.023) | (0.024) | (0.024) |
| municipalities ≤ 10,000 inhabitants | -0.112*** | -0.112*** | -0.156*** | -0.156*** | -0.125*** | -0.126*** | -0.066*** | -0.066*** | -0.095*** | -0.095*** |
|  | (0.008) | (0.008) | (0.017) | (0.017) | (0.017) | (0.017) | (0.017) | (0.017) | (0.017) | (0.017) |
| municipalities > 10,000 inhabitants | -0.021*** | -0.021*** | -0.056*** | -0.056*** | -0.033** | -0.033** | 0.014 | 0.014 | -0.004 | -0.004 |
|  | (0.008) | (0.008) | (0.016) | (0.016) | (0.016) | (0.016) | (0.016) | (0.016) | (0.016) | (0.016) |
| Italian region fixed effects | yes | yes | yes | yes | yes | yes | yes | yes | yes | yes |
| year fixed effects | yes | yes | no | no | no | no | no | no | no | no |
|  |  |  |  |  |  |  |  |  |  |  |
| Observations | 145,728 | 145,728 | 37,532 | 37,532 | 36,085 | 36,085 | 36,825 | 36,825 | 35,286 | 35,286 |
|  |  |  |  |  |  |  |  |  |  |  |

Source: Source: Multipurpose Survey on Households provided by [https://www.istat.it](https://www.istat.it/)
Notes: Ordered Probit coefficient estimates; robust standard errors in parentheses (data are unweighted). Significance: *** p<0.01, ** p<0.05, * p<0.10. The analyses are based on the full sample (employed, unemployed, or out of the labor force) where individuals below 18 years old are excluded. The outcome variable *trust in European parliament* is an ordinal variable, ranging from 0 (no trust at all) to 10 (complete trust). The dummy variable *online politics via social media* measures whether an individual does inquire about politics online through social media, such as Facebook or Twitter. The dummy variable *online politics w/o social media* measures whether an individual does inquire about politics online without using social media.

Table A.4.2

|  | (1) | (2) | (3) | (4) | (5) | (6) | (7) | (8) | (9) | (10) |
| --- | --- | --- | --- | --- | --- | --- | --- | --- | --- | --- |
|  | 2013 to 2016 | | 2013 | | 2014 | | 2015 | | 2016 | |
|  |  |  |  |  |  |  |  |  |  |  |
| online politics w/o social media | 0.045*** | 0.060*** | 0.092*** | 0.140*** | 0.035 | 0.047 | 0.045** | 0.028 | 0.006 | 0.026 |
|  | (0.011) | (0.019) | (0.021) | (0.039) | (0.022) | (0.039) | (0.021) | (0.038) | (0.022) | (0.040) |
| online politics via social media | -0.083*** | -0.012 | -0.050* | 0.063 | -0.078*** | 0.003 | -0.046* | 0.016 | -0.147*** | -0.102** |
|  | (0.013) | (0.024) | (0.028) | (0.053) | (0.028) | (0.050) | (0.026) | (0.046) | (0.025) | (0.046) |
| high school diploma | -0.211*** | -0.192*** | -0.231*** | -0.189*** | -0.177*** | -0.152*** | -0.251*** | -0.253*** | -0.185*** | -0.171*** |
|  | (0.011) | (0.016) | (0.022) | (0.031) | (0.022) | (0.031) | (0.021) | (0.031) | (0.022) | (0.032) |
| compulsory school | -0.300*** | -0.273*** | -0.328*** | -0.279*** | -0.307*** | -0.288*** | -0.295*** | -0.278*** | -0.267*** | -0.238*** |
|  | (0.013) | (0.016) | (0.025) | (0.032) | (0.026) | (0.032) | (0.025) | (0.032) | (0.026) | (0.033) |
| online politics w/o social media*h.s. diploma |  | -0.009 |  | -0.048 |  | -0.021 |  | 0.047 |  | -0.017 |
|  |  | (0.024) |  | (0.047) |  | (0.049) |  | (0.047) |  | (0.050) |
| online politics w/o social media*compulsory school |  | -0.026 |  | -0.091 |  | 0.024 |  | -0.008 |  | -0.041 |
|  |  | (0.033) |  | (0.064) |  | (0.068) |  | (0.065) |  | (0.069) |
| online politics via social media*h.s. diploma |  | -0.076** |  | -0.133** |  | -0.110* |  | -0.056 |  | -0.028 |
|  |  | (0.030) |  | (0.065) |  | (0.062) |  | (0.058) |  | (0.057) |
| online politics via social media*compulsory school |  | -0.184*** |  | -0.229** |  | -0.145 |  | -0.205** |  | -0.180** |
|  |  | (0.042) |  | (0.089) |  | (0.092) |  | (0.080) |  | (0.076) |
| women | 0.054*** | 0.054*** | 0.016 | 0.017 | 0.059*** | 0.059*** | 0.070*** | 0.069*** | 0.074*** | 0.073*** |
|  | (0.009) | (0.009) | (0.017) | (0.017) | (0.018) | (0.018) | (0.017) | (0.017) | (0.018) | (0.018) |
| 25-39 | -0.016 | -0.018 | -0.009 | -0.012 | 0.080* | 0.080* | -0.109** | -0.111** | -0.036 | -0.036 |
|  | (0.024) | (0.024) | (0.045) | (0.045) | (0.047) | (0.047) | (0.050) | (0.050) | (0.047) | (0.047) |
| 40-54 | 0.036 | 0.035 | 0.089* | 0.086* | 0.114** | 0.114** | -0.079 | -0.080 | 0.008 | 0.009 |
|  | (0.024) | (0.024) | (0.046) | (0.046) | (0.048) | (0.048) | (0.050) | (0.051) | (0.047) | (0.047) |
| 55-64 | 0.044* | 0.043* | 0.125** | 0.123** | 0.115** | 0.116** | -0.064 | -0.066 | -0.007 | -0.006 |
|  | (0.026) | (0.026) | (0.050) | (0.050) | (0.051) | (0.051) | (0.054) | (0.054) | (0.051) | (0.051) |
| 65+ | 0.085** | 0.083** | -0.054 | -0.057 | 0.329*** | 0.330*** | 0.021 | 0.019 | 0.020 | 0.019 |
|  | (0.039) | (0.039) | (0.081) | (0.081) | (0.077) | (0.077) | (0.079) | (0.079) | (0.077) | (0.077) |
|  |  |  |  |  |  |  |  |  |  |  |
| (continued on next page) | | | | | | | | | | |
|  | | | | | | | | | | |
|  | | | | | | | | | | |
|  | | | | | | | | | | |
|  | | | | | | | | | | |
|  | | | | | | | | | | |
|  | | | | | | | | | | |
|  | | | | | | | | | | |
|  | | | | | | | | | | |
| Table A.4.2 (continued) | | | | | | | | | | |
|  |  |  |  |  |  |  |  |  |  |  |
| married | 0.045*** | 0.045*** | 0.017 | 0.018 | 0.027 | 0.027 | 0.091*** | 0.091*** | 0.051** | 0.050** |
|  | (0.013) | (0.013) | (0.025) | (0.025) | (0.026) | (0.026) | (0.026) | (0.026) | (0.025) | (0.025) |
| couple with children | -0.091*** | -0.091*** | -0.065** | -0.066** | -0.085*** | -0.084*** | -0.118*** | -0.118*** | -0.097*** | -0.096*** |
|  | (0.015) | (0.015) | (0.031) | (0.031) | (0.031) | (0.031) | (0.031) | (0.031) | (0.030) | (0.030) |
| couple without children | -0.112*** | -0.112*** | -0.077** | -0.079** | -0.119*** | -0.117*** | -0.188*** | -0.187*** | -0.069* | -0.068* |
|  | (0.018) | (0.018) | (0.036) | (0.036) | (0.036) | (0.036) | (0.036) | (0.036) | (0.036) | (0.036) |
| single-parent father | -0.074** | -0.073** | -0.029 | -0.026 | -0.150** | -0.151*** | -0.044 | -0.044 | -0.059 | -0.060 |
|  | (0.031) | (0.031) | (0.066) | (0.067) | (0.058) | (0.058) | (0.062) | (0.062) | (0.061) | (0.061) |
| single-parent mother | -0.080*** | -0.079*** | -0.079** | -0.078** | -0.100*** | -0.099*** | -0.075** | -0.074** | -0.066* | -0.065* |
|  | (0.018) | (0.018) | (0.037) | (0.037) | (0.037) | (0.037) | (0.035) | (0.035) | (0.036) | (0.036) |
| municipalities ≤ 10,000 inhabitants | -0.143*** | -0.142*** | -0.165*** | -0.164*** | -0.135*** | -0.135*** | -0.146*** | -0.146*** | -0.121*** | -0.120*** |
|  | (0.013) | (0.013) | (0.026) | (0.026) | (0.026) | (0.026) | (0.026) | (0.026) | (0.027) | (0.027) |
| municipalities > 10,000 inhabitants | -0.030** | -0.030** | -0.047* | -0.046* | -0.022 | -0.021 | -0.043* | -0.043* | -0.003 | -0.002 |
|  | (0.012) | (0.012) | (0.024) | (0.024) | (0.024) | (0.024) | (0.025) | (0.025) | (0.026) | (0.026) |
| Italian region fixed effects | yes | yes | yes | yes | yes | yes | yes | yes | yes | yes |
| year fixed effects | yes | yes | no | no | no | no | no | no | no | no |
|  |  |  |  |  |  |  |  |  |  |  |
| Observations | 61,299 | 61,299 | 15,718 | 15,718 | 15,041 | 15,041 | 15,487 | 15,487 | 15,053 | 15,053 |
|  |  |  |  |  |  |  |  |  |  |  |

Source: Source: Multipurpose Survey on Households provided by [https://www.istat.it](https://www.istat.it/)
Notes: Ordered Probit coefficient estimates; robust standard errors in parentheses (data are unweighted). Significance: *** p<0.01, ** p<0.05, * p<0.10. The analyses are based on the sample of employed workers, where individuals below 18 years old are excluded. The outcome variable *trust in European parliament* is an ordinal variable, ranging from 0 (no trust at all) to 10 (complete trust). The dummy variable *online politics via social media* measures whether an individual does inquire about politics online through social media, such as Facebook or Twitter. The dummy variable *online politics w/o social media* measures whether an individual does inquire about politics online without using social media.

Table A.5

|  | (1) | (2) | (3) | | (4) | | (5) | | (6) | | (7) | | (8) | |  |
| --- | --- | --- | --- | --- | --- | --- | --- | --- | --- | --- | --- | --- | --- | --- | --- |
|  | Full sample | | | | | | Sample of employed | | | | | | | |  |
|  | w/o computer skills | | with computer skills | | | | w/o computer skills | | | | with computer skills | | | |  |
| online politics w/o social media | 0.024** | 0.027 | | 0.010 | | 0.019 | | 0.024 | | 0.026 | | 0.014 | | 0.023 | |
|  | (0.012) | (0.022) | | (0.013) | | (0.024) | | (0.015) | | (0.027) | | (0.017) | | (0.029) | |
| online politics via social media | -0.069*** | -0.034 | | -0.089*** | | -0.054* | | -0.099*** | | -0.045 | | -0.106*** | | -0.055 | |
|  | (0.014) | (0.027) | | (0.015) | | (0.028) | | (0.018) | | (0.032) | | (0.020) | | (0.034) | |
| high school diploma | -0.190*** | -0.182*** | | -0.173*** | | -0.163*** | | -0.218*** | | -0.211*** | | -0.211*** | | -0.202*** | |
|  | (0.012) | (0.017) | | (0.013) | | (0.018) | | (0.015) | | (0.022) | | (0.016) | | (0.023) | |
| compulsory school | -0.261*** | -0.251*** | | -0.223*** | | -0.210*** | | -0.280*** | | -0.257*** | | -0.253*** | | -0.226*** | |
|  | (0.013) | (0.016) | | (0.014) | | (0.018) | | (0.018) | | (0.023) | | (0.021) | | (0.026) | |
| online politics w/o social media*h.s. diploma |  | -0.012 | |  | | -0.019 | |  | | 0.015 | |  | | 0.005 | |
|  |  | (0.027) | |  | | (0.029) | |  | | (0.034) | |  | | (0.036) | |
| online politics w/o social media*compulsory school |  | 0.032 | |  | | 0.024 | |  | | -0.022 | |  | | -0.037 | |
|  |  | (0.035) | |  | | (0.037) | |  | | (0.047) | |  | | (0.050) | |
| online politics via social media*h.s. diploma |  | -0.023 | |  | | -0.019 | |  | | -0.042 | |  | | -0.037 | |
|  |  | (0.032) | |  | | (0.033) | |  | | (0.041) | |  | | (0.042) | |
| online politics via social media*compulsory school |  | -0.120*** | |  | | -0.132*** | |  | | -0.191*** | |  | | -0.190*** | |
|  |  | (0.041) | |  | | (0.043) | |  | | (0.055) | |  | | (0.058) | |
| women | 0.049*** | 0.049*** | | 0.049*** | | 0.049*** | | 0.072*** | | 0.071*** | | 0.068*** | | 0.067*** | |
|  | (0.008) | (0.008) | | (0.008) | | (0.008) | | (0.012) | | (0.012) | | (0.013) | | (0.013) | |
| 25-39 | -0.224*** | -0.226*** | | -0.220*** | | -0.222*** | | -0.069** | | -0.070** | | -0.071** | | -0.072** | |
|  | (0.017) | (0.017) | | (0.018) | | (0.018) | | (0.034) | | (0.034) | | (0.035) | | (0.035) | |
| 40-54 | -0.197*** | -0.198*** | | -0.199*** | | -0.200*** | | -0.031 | | -0.032 | | -0.046 | | -0.045 | |
|  | (0.018) | (0.018) | | (0.019) | | (0.019) | | (0.034) | | (0.035) | | (0.036) | | (0.036) | |
| 55-64 | -0.207*** | -0.208*** | | -0.199*** | | -0.200*** | | -0.031 | | -0.032 | | -0.038 | | -0.038 | |
|  | (0.020) | (0.020) | | (0.022) | | (0.022) | | (0.037) | | (0.037) | | (0.039) | | (0.039) | |
| 65+ | -0.090*** | -0.092*** | | -0.082*** | | -0.084*** | | 0.023 | | 0.021 | | 0.003 | | 0.003 | |
|  | (0.020) | (0.020) | | (0.022) | | (0.022) | | (0.055) | | (0.055) | | (0.059) | | (0.059) | |
|  |  |  | |  | |  | |  | |  | |  | |  | |
| (continued on next page) | | | | | | | | | | | | | | | |
|  |  |  | |  | |  | |  | |  | |  | |  | |
|  |  |  | |  | |  | |  | |  | |  | |  | |
|  |  |  | |  | |  | |  | |  | |  | |  | |
| Table A.5 (continued) | | | | | | | | | | | | | | | |
|  |  |  | |  | |  | |  | |  | |  | |  | |
| married | 0.054*** | 0.055*** | | 0.065*** | | 0.065*** | | 0.070*** | | 0.069*** | | 0.077*** | | 0.076*** | |
|  | (0.014) | (0.014) | | (0.014) | | (0.014) | | (0.018) | | (0.018) | | (0.019) | | (0.019) | |
| couple with children | -0.068*** | -0.067*** | | -0.075*** | | -0.074*** | | -0.108*** | | -0.107*** | | -0.112*** | | -0.112*** | |
|  | (0.016) | (0.016) | | (0.016) | | (0.016) | | (0.021) | | (0.021) | | (0.022) | | (0.022) | |
| couple without children | -0.078*** | -0.078*** | | -0.087*** | | -0.087*** | | -0.126*** | | -0.126*** | | -0.136*** | | -0.135*** | |
|  | (0.017) | (0.017) | | (0.018) | | (0.018) | | (0.026) | | (0.026) | | (0.026) | | (0.026) | |
| single-parent father | -0.001 | -0.002 | | -0.008 | | -0.008 | | -0.051 | | -0.051 | | -0.056 | | -0.057 | |
|  | (0.030) | (0.030) | | (0.032) | | (0.032) | | (0.043) | | (0.043) | | (0.045) | | (0.045) | |
| single-parent mother | -0.053*** | -0.053*** | | -0.056*** | | -0.056*** | | -0.071*** | | -0.070*** | | -0.074*** | | -0.074*** | |
|  | (0.016) | (0.016) | | (0.017) | | (0.017) | | (0.025) | | (0.025) | | (0.026) | | (0.026) | |
| municipalities ≤ 10,000 inhabitants | -0.082*** | -0.082*** | | -0.088*** | | -0.087*** | | -0.135*** | | -0.134*** | | -0.135*** | | -0.134*** | |
|  | (0.012) | (0.012) | | (0.013) | | (0.013) | | (0.019) | | (0.019) | | (0.019) | | (0.019) | |
| municipalities > 10,000 inhabitants | 0.003 | 0.004 | | -0.001 | | -0.001 | | -0.025 | | -0.024 | | -0.027 | | -0.027 | |
|  | (0.011) | (0.011) | | (0.012) | | (0.012) | | (0.018) | | (0.018) | | (0.018) | | (0.018) | |
| proxies for computer skills | no | no | yes | | yes | | no | | no | | yes | | yes | |  |
| Italian region fixed effects | yes | yes | yes | | yes | | yes | | yes | | yes | | yes | |  |
| year fixed effects | yes | yes | yes | | yes | | yes | | yes | | yes | | yes | |  |
|  |  |  |  | |  | |  | |  | |  | |  | |  |
| Test for joint significance of computer skills | | | | | | | | | | | | | | |  |
| *F*-test |  |  | 93.24*** | | 93.58*** | |  | |  | | 51.58*** | | 52.17*** | |  |
| Observations | 72,111 | 72,111 | 66,812 | | 66,812 | | 30,540 | | 30,540 | | 28,402 | | 28,402 | |  |
|  |  |  |  | |  | |  | |  | |  | |  | |  |

Source: Source: Multipurpose Survey on Households provided by <https://www.istat.it>
Notes: Ordered Probit coefficient estimates; robust standard errors in parentheses (data are unweighted). Significance: *** p<0.01, ** p<0.05, * p<0.10. The analyses are based on the full sample (employed, unemployed, or out of the labor force) and the sample of employed workers, pooled over 2015 and 2016, where individuals below 18 years old are excluded. The outcome variable *trust in European parliament* is an ordinal variable, ranging from 0 (no trust at all) to 10 (complete trust). The dummy variable *online politics via social media* measures whether an individual does inquire about politics online through social media, such as Facebook or Twitter. The dummy variable *online politics w/o social media* measures whether an individual does inquire about politics online without using social media. The proxy variables for self-assessed computer skills are derived from various operations that were performed in the last 12 months: transfer files between computers and/or other devices such as digital camera, cell phone, or MP3 player (yes/no), install software or applications (yes/no), change the settings of any software, including operating systems or security programs (yes/no), Connect and install peripherals like printers or modems (yes/no), compress or zip files (yes/no), copy or move a file or folder (yes/no), use software for word processing like e.g. Microsoft Word (yes/no), use “copy and paste” to copy or move information within a document (yes/no), create presentations or documents that include texts, images, graphics, tables (yes/no), use spreadsheets for calculation like e.g. Microsoft Excel (yes/no), use the advanced functions of the spreadsheets for calculation to organize and analyze data like e.g. sort, filter, use formulas, create graphics (yes/no), use software to edit photos, videos, audio files (yes/no) and write code in a programming language (yes/no); these variables are available in this form only in 2015 and 2016.

Table A.6

|  | (1) | (2) | (3) | (4) | (5) | (6) | (7) | (8) |
| --- | --- | --- | --- | --- | --- | --- | --- | --- |
|  | Labor force sample | | | | Sample of employed | | | |
|  | Standard ordered probit | | IV ordered probit | | Standard ordered probit | | IV ordered probit | |
|  | Compulsory education | Higher education | Compulsory education | Higher education | Compulsory education | Higher education | Compulsory education | Higher education |
| online politics w/o social media | 0.051* | 0.075*** | 0.01 | -0.002 | 0.03 | 0.073*** | 0.048 | -0.06 |
|  | (0.028) | (0.013) | (0.130) | (0.128) | (0.031) | (0.014) | (0.143) | (0.134) |
| online politics via social media | -0.171*** | -0.033** | -0.406*** | 0.048 | -0.203*** | -0.033** | -0.495*** | 0.053 |
|  | (0.034) | (0.015) | (0.134) | (0.070) | (0.038) | (0.017) | (0.139) | (0.075) |
| control variables | yes | yes | yes | yes | yes | yes | yes | yes |
| Italian region fixed effects | yes | yes | yes | yes | yes | yes | yes | yes |
| year fixed effects | yes | yes | yes | yes | yes | yes | yes | yes |
|  |  |  |  |  |  |  |  |  |
| *F* test (dep. var. -> online politics w/o social media) | | | | | | | | |
|  |  |  | 439.47*** | 533.09*** |  |  | 290.87*** | 371.78*** |
| *F* test (dep. var. -> online politics via social media) | | | | | | | | |
|  |  |  | 355.34*** | 872.19*** |  |  | 242.67*** | 606.50*** |
| Observations | 19,565 | 38,771 | 19,495 | 38,715 | 14,208 | 31,373 | 14,170 | 31,336 |
|  |  |  |  |  |  |  |  |  |

Source: Source: Multipurpose Survey on Households provided by [https://www.istat.it](https://www.istat.it/)
Notes: Standard and IV Ordered Probit coefficient estimates; robust standard errors in parentheses (data are unweighted). The IV ordered probit estimation involves two first-stage probit regressions. Significance: *** p<0.01, ** p<0.05, * p<0.10. The analyses are based on the labor-force sample and the sample of employed workers, pooled over 2014, 2015 and 2016, where individuals below 18 years old are excluded. The outcome variable *trust in European parliament* is an ordinal variable, ranging from 0 (no trust at all) to 10 (complete trust). The dummy variable *online politics via social media* measures whether an individual does inquire about politics online through social media, such as Facebook or Twitter. The dummy variable *online politics w/o social media* measures whether an individual does inquire about politics online without using social media. The control variables are sex, age groups, married, household type, and urban level. The instrumental variables are DSL connexion (yes/no), smartphone connexion (yes/no), SIM/USB connexion (yes/no) and ISDN connexion (yes/no); these four variables are available in this form only in 2014, 2015 and 2016.
